# Population-Based Cancer Registration in Sub-Saharan Africa: Its Role in Research and Cancer Control

**DOI:** 10.1200/GO.20.00294

**Published:** 2020-11-12

**Authors:** Abidemi Emmanuel Omonisi, Biying Liu, Donald Maxwell Parkin

**Affiliations:** ^1^ Department of Anatomic Pathology, Ekiti State University, Ado-Ekiti, Nigeria; ^2^ Ekiti Cancer Registry, Ekiti State University Teaching Hospital, Ado-Ekiti, Nigeria; ^3^ African Cancer Registry Network, Oxford, United Kingdom; ^4^ Nuffield Department of Population Health, University of Oxford, Oxford, United Kingdom; ^5^ International Agency for Research on Cancer, Lyon, France

## INTRODUCTION

Cancer disproportionately affects patients in low- and middle-income countries, with 70% of newly diagnosed cancer cases occurring in low- and middle-income countries where the survival rate of cancer is 30% to 50% lower than that of high-income countries.^1^ Although Africa only accounts for approximately 6% of the world cancer burden, it is not a rare disease on the continent, as is sometimes supposed. The economic and social burdens it brings upon the population are enormous. To overcome this rapidly rising problem, countries in sub-Saharan Africa (SSA) urgently need rational national cancer control planning.

**CONTEXT**

**Key Objective**
The aim of this article is to report on the current status of cancer registration in sub-Saharan Africa and to showcase the various critical strategic roles and applications of cancer registries in cancer research and cancer control programs.
**Knowledge Generated**
Countries in sub-Saharan Africa need a working cancer control program to help battle the ever-increasing burden of cancer. A rational cancer control program must be built on recent, accurate population-based data on incidence, survival, treatment, and outcome from within its nation.
**Relevance**
Cancer registries can play an important role in the evaluation and monitoring of screening programs aimed at detecting preinvasive conditions. The quality and quantity of the data generated by these registries have been used to support research and statistics and have been disseminated globally.

The aim of this article is to report on the current status of cancer registration in SSA, particularly the pivotal role of the African Cancer Registry Network (AFCRN) in serving as the regional hub for the International Agency for Research on Cancer (IARC) in coordinating cancer registration in the region. We showcase the various critical strategic roles and applications of cancer registries in cancer research and cancer control programs

### Cancer Surveillance in Africa

Information on cancer burden in a country is a crucial basis for building and monitoring any rational cancer control programs. Data needed may be available from death registration systems, which provide data on mortality rates by cause of death. Such statistics have been available for high-income countries for decades; however, the absence of comprehensive and accurate death registration systems is a major defect in almost all SSA countries—only approximately 0.25% of the SSA population is covered by accurate death registration systems.^2^ This means that the only available alternative for obtaining real information on the occurrence of cancer is through cancer registration

### Cancer Registries

Cancer registries provide for the systematic collection, storage, analysis, interpretation, and reporting of data on patients with cancer.^3^ There are three types of cancer registries: hospital-based, specialized, and—the most complex—population-based cancer registries.

Hospital-based cancer registries are primarily institution based and are involved with recording information on patients with cancer attending a particular hospital. The main purpose of these registries is to contribute to patient care by providing readily accessible information on patients with cancer and treatment received and its result.^4^ Specialized registries collect and maintain data on a particular type of cancer—for example, the Gilda Radner Familial Ovarian Cancer Registry,^5^ which collects cancer information on families with ovarian cancer.

Population-based cancer registries (PBCRs) seek to collect data on all new cases of cancer occurring in a well-defined population. Usually, the population consists of the residents in a particular geographic region.^4^ The main objective of population-based cancer registries is to provide statistics on the occurrence of cancer in that population and to provide a framework for assessing and controlling the impact of cancer in the community. The emphasis here is on epidemiology and public health. Hospital-based and special registries may contribute data to PBCRs, but they have fundamental differences in their core functions and are not a substitute for them.^6^

### Role of Cancer Registries

It has been accepted generally that the PBCR has more of a back room role than a front-line role in cancer control. Its particular responsibilities lie in the description of cancer patterns, care, and outcome; in monitoring these variables in relation to control activities; and in providing a research database—often for others to use.^7^ The original function of PBCRs was to calculate the rates of incidence so that the risk of various cancers between populations could be compared. In addition, the activities of PBCRs have expanded to include studies of cancer cause and prevention.^7^

Uses of data from PBCRs include:

1. To describe the extent and nature of cancer burden in the community and assist in the establishment of public health priorities
2. As a source of material for etiologic studies
3. To help in planning, monitoring, and assessing the effectiveness of national cancer control programs.

## CANCER REGISTRATION IN AFRICA

### Geographic Location and Peculiarities of SSA

SSA is geographically the region of the continent that lies south of the Sahara. The United Nations Development Program lists 46 of Africa’s 54 countries as sub-Saharan.^8,9^ These countries share many characteristics and challenges related, but are not limited, to the possession of one of the weakest public health systems in the world. A large proportion inhabitants lack adequate access to basic health care, resulting in poor health indices and extremely high mortality rates.^10-12^ The region has one of the highest prevalence of HIV/AIDS in the world,^13^ and the subsequent proportion of AIDS-associated malignancies is significantly higher compared with the rest of the world.^14^ Most parts of SSA are warm and humid, which explains the preponderance of infectious diseases in this part of the world.^15^ Old diseases—familiar problems in the region, including malaria^16^ and tuberculosis^17^—coexist with new emerging and re-emerging diseases, such as Ebola,^18^ Lassa fever,^19^ and cholera.^20^ In addition to these infectious diseases are noncommunicable diseases, such as hypertension,^21^ diabetes,^22^ and accidents and violence,^23^ as well as cancer.

### Cancer Registration in Africa

Some information on cancer patterns in Africa was available in the first half of the 20th century though the work of pioneering researchers, who published statistics gleaned from individual hospitals and clinics, and the first population-based registries were founded in the 1950s.^24^ Unfortunately, in the decades that followed, progress was slow. For the period covered by Cancer Incidence in Five Continents Volume V (1978 to 1982) there was no representative from Africa. A slow rebirth of cancer registration has been taking place since the 1980s.^25^ Reasons for this slow progress have been described many times: insufficient coordination of data sources, lack of adequately trained technical staff, lack of up-to-date population census figures, poor health infrastructure, lack of political will, and poor procurement systems marred by mismanagement and corruption.^25-27^ Even when a registry is operating, it faces countless challenges, such as generally poor health care infrastructure,^25^ especially in rural areas^28^; lack of a regular and accurate census program^29-31^; absence of vital statistics; the inability to ensure that all new cases were identified and captured in the databases; lack of adequately trained personnel; and lack of cooperation from other data sources. Computer-based medical information systems remain underdeveloped, which means that any data linkage or observation of patients with cancer are tiresome and expensive—patient observation for calculation of survival involves actively tracing patients and their family by mail, telephone, or home visit.^32^ Passive follow up is feasible only in the few countries where a reliable death registration system exists.

Despite these problems, there has been a slow growth in the number of cancer registries and, just as importantly, their quality. In Nigeria, for example, the National System of Cancer Registries was established in 2009,^33^ with support from the Nigerian government, and has facilitated many training courses and consultancy visits to support the development of new population-based registries. A 2014 survey identified 25 functioning PBCRs, and the 23 that took part covered a total population of 90.7 million people, some 10.5% of the SSA population.^34^

### Roles of PBCR in SSA

#### National cancer control program.

A national cancer control program (NCCP) is a systematic, equitable, and evidence-based program that, if well implemented, has the capability to reduce the incidence of certain important cancers, such as cancer of cervix, and to improve survival and quality of life for all patients with cancer.^35^ A rational NCCP can only be built upon and be monitored using accurate evidence provided by PBCRs from within its own country.^24,36^ Sadly, most SSA countries still have not developed a comprehensive NCCP—a 2014 survey by the US National Cancer Institute found that only 11 countries in Africa had a current NCCP.^37^

#### Health care planning and monitoring.

Cancer registries provide statistical information on the number of cases in the population. Accurate information on cancer occurrence is important for fixing priorities and targeting cancer control activities. Annual numbers of incident cases provide an indication of the resources needed for primary treatment, and the number of prevalent cases describes how many people are in need of regular long-term follow up. Information from cancer registries may be used for the planning and establishment of cancer treatment and care facilities directed toward various types of cancer. Geographic differences in cancer occurrence should be taken into account, as well as changes over time in different cancers—these can be used to make projections of future incidence rates, caseloads, and need for treatment facilities. Cancer incidence information has been used for the planning of radiotherapy services in the United Kingdom.^38^ Unfortunately, a similar projection has not been performed in the SSA region despite cancer registry data being improved and made more available for governments and other stakeholders. In one study, only 23 of 56 countries in Africa, concentrated in the southern and northern extremes of the continent, were shown to have megavoltage therapy.^39^

Linking information on the incidence of cancer in the population with data on the prevalence of risk factors allows for the estimation of the population-attributable fraction—that is, the proportion of cancer cases that might be prevented if the risk factor was eliminated (or reduced to a lower level). This is clearly an important exercise in estimating the potential impact of preventive interventions—how much cancer might be prevented by our efforts. This type of exercise may be done for specific national populations—for example, the fraction of cancers in Nigeria that are caused by alcohol^40^ or being overweight and obese^41^—or for the continent-wide burden of cancer as a result of infectious agents.^42^

Stage at diagnosis is an important statistic to document. For the planner, it is an objective indicator of how early cancer is being diagnosed and thus indicates where improvements can be made. This may be via programs of early diagnosis (eg, by education of the population in recognizing cancer early and seeking help) or population screening, or by ensuring that the medical system provides for the prompt referral and management of patients with symptoms. Stage is also important in interpreting the outcome of cancer: the proportion of patients who will survive their disease. Clearly, the prospects of cure are much better when cancer is diagnosed early. Increasing efforts are being made to collect information on stage at diagnosis by cancer registries in Africa.^43^ Until recently, there was relatively little information for unselected populations; most of the data available are from clinical case series.^44^

Documenting survival at 1, 3, and 5 years after diagnosis is a standard practice of registries in high-income countries, but this has been difficult to achieve in Africa, because of the difficulties of tracing patients with cancer once they are discharged from the hospital, as described earlier. AFCRN has made a concerted effort to improve the situation, and progressively more information on cancer survival in adults^32,45^ and children^46^ in African populations is becoming available.

Primary prevention programs include tobacco control, as in most high-income countries, but in Africa, the most important interventions will aim to reduce the burden of infection-associated cancers. Vaccination programs against hepatitis B—the most important cause of liver cancer—have been implemented since the early 1990s in many countries, and vaccination against human papillomavirus is being rolled out currently.^47^ Registries will have an important role in monitoring the incidence of cancers associated with these viruses. An example of data from a PBCR used to monitor the effects of a preventive intervention—in the setting of a major epidemiologic research study—is The Gambia Hepatitis Intervention Study, a large-scale vaccination trial in The Gambia instituted in July 1986.^48^ This was designed as an intervention trial by WHO to reverse the high incidence of hepatocellular carcinoma, the results of which would be monitored by The Gambia cancer registry.

The purpose of population-based programs of early diagnosis and screening is the detection of early invasive cancers, reduction in mortality from a particular cancer, and improved quality of life through the use of less toxic treatments with early detection. Cancer registries can play an important role in the evaluation and monitoring of screening programs aimed at detecting preinvasive conditions. Examination of asymptomatic persons to detect cancer at early stages is becoming increasingly important in the control of certain cancers, such as cervical cancer.^49^ Cervical cancer screening programs have been reported to lower the incidence of cervical cancer in some parts of the world.^50^

Similar initiatives could be implemented and extended to most preventable cancers in the region.

#### Epidemiologic research.

PBCRs are important sources of educational resources for researchers as they provide unique information on the distribution of cancer in a well-defined geographic population. Researchers can use the available data to elucidate the determinants of cancer in a particular population. As described above, the AFCRN database can be used to conduct research and for planning and implementation of cancer control programs and treatments in the entire region and in an individual country. The basic role of a cancer registry is to provide statistical information on cancer occurrence and outcomes. Registries are widely used to study the incidence of different cancers within registry populations^51,52^ or in subgroups of the population,^53,54^ as well as the regional distribution of specific cancers.^55,56^ Temporal trends in specific registry populations,^57^ or for individual cancers in multiple registries continent wide,^58,59^ are particularly valuable in following results of the changing profiles of environmental exposures.

Use of cancer registries in formal epidemiologic studies in Africa is much less well developed than in high-income settings. They have been used, however, to detect the occurrence of cancer in cohorts of individuals with specific exposures—for example, infection with Epstein-Barr virus^60^ or HIV.^61^

## STRENGTHENING CANCER REGISTRATION IN SSA: THE ROLE OF AFCRN

**FIG 1:**
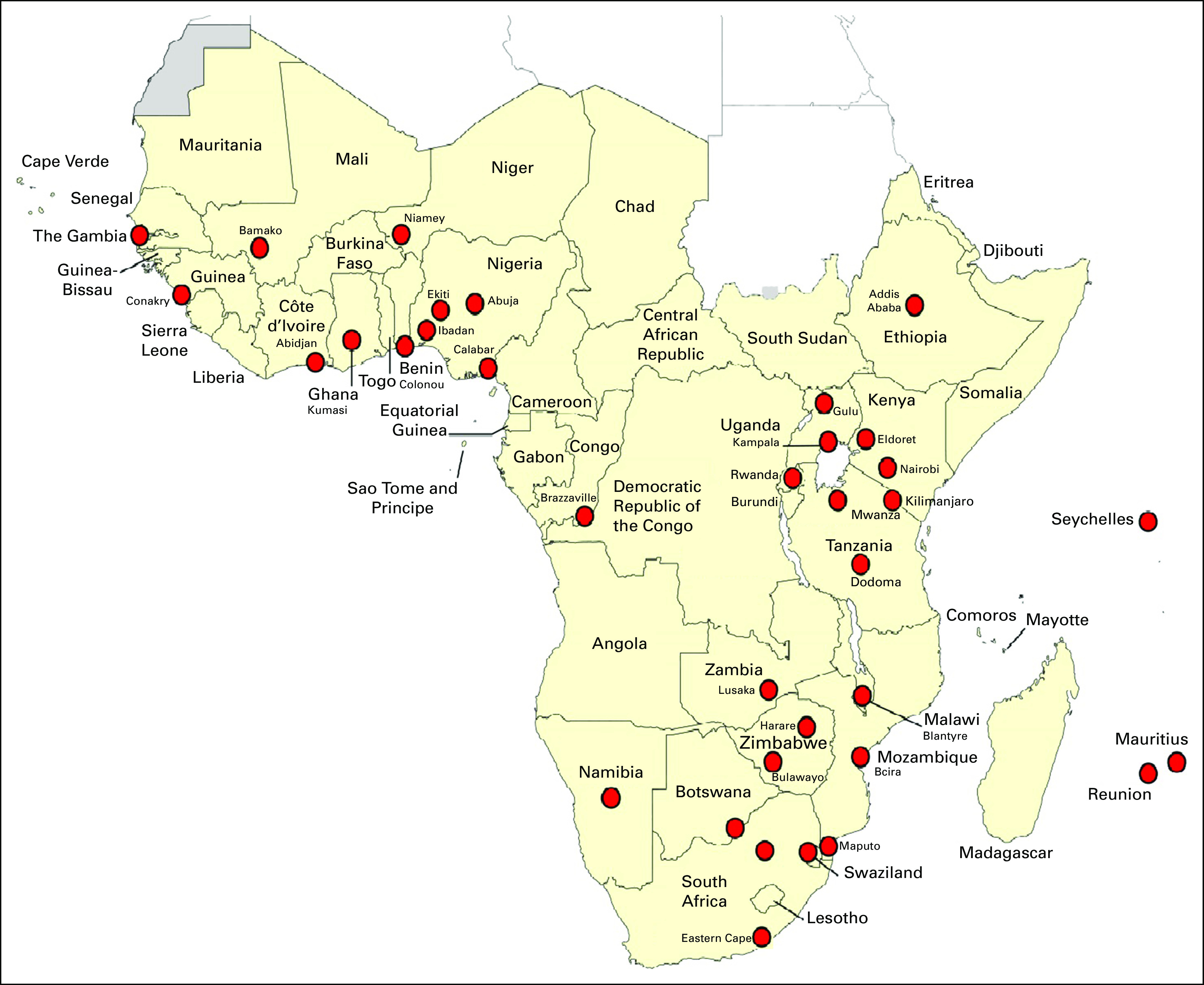
Membership of the African Cancer Registry Network in sub-Saharan Africa, September 2020.

AFCRN was founded in March 2012 as a consortium of all the genuine functioning PBCRs in SSA. By 2020, its membership had grown from 16 member registries in 12 countries to 35 member registries in 25 countries^62^ (Fig 1).

In September 2012, the IARC designated AFCRN as its regional hub for cancer registration in SSA in the framework of its Global Initiative for Cancer Registry Development in Low- and Middle-Income Countries.^63^ The overall goal of the hub is to extend and strengthen the capacity for cancer registration on the continent by providing training courses and technical advice, advocacy, and political lobbying. It is a platform for international networking and research. The increased availability of information on cancer incidence and mortality, treatment, and stage at diagnosis, as well as risk factors for cancer, allows for the systematic and rational development of cancer control programs within the registries’ own countries.

AFCRN has been active in the fields of training, technical support, research coordination, networking, and advocacy. The coordination center, led by a senior scientist assisted by an administrator, exists to raise funds, coordinate activities on the network, and to pave the way for future developments in registration on the continent. An advisory committee consisting of representatives from WHO (AFRO), International Association of Cancer Registries, Union for International Cancer Control, and IARC oversees AFCRN’s plans and actions. Over the years, financial support has been received from a variety of organizations, as well as foundations of international pharmaceutical companies, which have helped strengthen the capacity of many member registries. AFCRN research projects are often led by young scientists from Africa, which are funded by various research institutes, such as IARC, the American Cancer Society, the Union for International Cancer Control, the University of Halle, and the University of Oxford (Africa-Oxford Initiative).

One of AFCRN’s significant achievements is that it has created a unique database for cancer in SSA. The database contains data from all member registries from the year when they started population-based registration. The database has facilitated numerous research studies and provides the material for monographs on cancer in Africa,^63,64^ as well as for IARC’s national estimates of cancer incidence, mortality, and prevalence (GLOBOCAN estimates).^1^ The database is accessible via the AFCRN Research Committee (procedure is available online^65^).

The data are not perfect, nor is AFCRN. Dedicated African registrars, doctors, nurses, and policymakers, together with international researchers, are working tirelessly to improve the situation. It is a battle worth fighting, they believe.

## THE WAY AHEAD

Of the 46 countries of the SSA, only 25 have population-based registries, the data for which can be used to make some sort of estimate of the national cancer profile. Apart from island populations, like Seychelles, Mauritius, and Reunion, these registries cover limited populations, nearly all of which are urban, from which the national profile has to be deduced. For the remaining 21 countries, estimation of the cancer profile has to be based on the picture in neighboring countries or some sort of simulation modeling.^66^ The availability of modeled estimates of morbidity and mortality seems reassuring; policymakers get a false impression of certainty about health status and trends, and this detracts from making much needed investments in improving data collection and analytical capacity within countries.^67^

Although the cancer registry is an essential part of any rational program of cancer control,^68^ in most SSA countries, there is an insufficient or no governmental budget for cancer registries. That is because health care policymakers in SSA countries are faced with numerous challenges, but also because cancer registries do not show any immediate impact on cancer prevention so that their necessity is not always recognized and appreciated. Historically, registries have worked in isolation, often operating thanks to the supervision and support of one or two keen medical doctors. Sometimes they are even independent of health departments or universities. Registries have been supported by a wide variety of funding agencies.^34^ In fact, PBCRs are not expensive. A recent study estimated the cost of cancer registration at the population level as 1 to 2 cents per person in three countries studied, Kenya, Uganda, and Zimbabwe.^69^

Despite all the problems mentioned, thanks to the efforts of international organizations, such as WHO and IARC; SSA governments; AFCRN; and, most importantly, the individual cancer registry staff, the situation is changing remarkably. The number of functioning PBCRs has been gradually increasing in all regions of SSA. In countries like Nigeria, Kenya, Tanzania, Uganda, and Zimbabwe, where there are multiple cancer registries, they have become indispensable. These registries submit their cancer reports to the central government regularly and some registry members sit on the NCCP board for making policies for their countries.

Several specially designed international research projects, such as SurvCan 3 (led by IARC) and Treatment and Follow Up (led by University of Halle), have provided additional resources and expertise to conduct studies aimed at determining the survival and treatment received by patients with cancer. Numerous research papers on trends in cancer incidence, led by young African researchers using AFCRN data, have been published in international peer-reviewed journals. A kernel of experienced cancer registry pioneers and registrars have formed a unique team of trainers for teaching cancer registration methods.

Countries such as Kenya, Tanzania, Eswatini, Cote d’Ivoire, and South Africa already have their own national cancer control planning based on their own data, and some of their strategies are being implemented. Improvements in the health care system for patients with cancer will be visible in the next 5 years.

Countries in SSA need a working cancer control program to help battle the ever-increasing burden of cancer. A rational cancer control program must be built upon recent, accurate population-based data on incidence, survival, treatment, and outcome from within the nation it serves. A functioning cancer registration program is the unique way to obtain such data. Estimates—based on opaque simulation models—should never be a substitute for actual data,^70^ especially in monitoring the effectiveness of cancer control measures. There is an urgent need to increase investment in primary data collection, especially in low- and middle-income countries, to reduce the reliance on complex statistical models.^71^

The two major common challenges confronting all PBCRs in the SSA are the lack of funding and poor infrastructure to support cancer registration. We recommend that resources for the development of cancer registries be prioritized urgently by the various governments in SSA. This is important as the current overdependence on donor agencies may not be sustainable in view of the emerging global financial crisis.

For nearly a decade, AFCRN has been actively involved in providing needed mentorship, establishing more PBCRs, building capacity, providing funding, and initiating cancer research across the various PBCRs in SSA. PBCRs are no longer the elephant in the room in SSA as a result of the various initiatives by AFCRN which have yielded various improvements in cancer registration on the continent; however, there is still much room for improvement in cancer registration on the continent. With increasing awareness and efforts made across the health care system within countries, marked improvement continues.
